# Quantitative gait analysis and prediction using artificial intelligence for patients with gait disorders

**DOI:** 10.1038/s41598-023-49883-8

**Published:** 2023-12-28

**Authors:** Nawel Ben Chaabane, Pierre-Henri Conze, Mathieu Lempereur, Gwenolé Quellec, Olivier Rémy-Néris, Sylvain Brochard, Béatrice Cochener, Mathieu Lamard

**Affiliations:** 1 LaTIM UMR 1101 Laboratory, Inserm, Brest, France; 2 Western Brittany University, Brest, France; 3 IMT Atlantique, Brest, France; 4 University Hospital of Brest, Brest, France

**Keywords:** Computer science, Quality of life, Data mining

## Abstract

Quantitative Gait Analysis (QGA) is considered as an objective measure of gait performance. In this study, we aim at designing an artificial intelligence that can efficiently predict the progression of gait quality using kinematic data obtained from QGA. For this purpose, a gait database collected from 734 patients with gait disorders is used. As the patient walks, kinematic data is collected during the gait session. This data is processed to generate the Gait Profile Score (GPS) for each gait cycle. Tracking potential GPS variations enables detecting changes in gait quality. In this regard, our work is driven by predicting such future variations. Two approaches were considered: signal-based and image-based. The signal-based one uses raw gait cycles, while the image-based one employs a two-dimensional Fast Fourier Transform (2D FFT) representation of gait cycles. Several architectures were developed, and the obtained Area Under the Curve (AUC) was above 0.72 for both approaches. To the best of our knowledge, our study is the first to apply neural networks for gait prediction tasks.

## Introduction

Gait disorders are described as any deviation from normal walking or gait^1^. Their prevalence among adults rises with age. In the elderly population over the age of 70 years, they are present in approximately 35% of patients^2,3^ and in 72% of patients over 80 years^2^. These statistics take into account whether such disorders result from neurological etiologies or not, which can be determined through laboratory work, clinical presentation, and diagnostic testing^2^. In fact, gait disorders etiologies include neurological conditions (e.g., sensory or motor impairments), orthopedic abnormalities (e.g., osteoarthritis and skeletal deformities), and medical conditions (e.g., heart failure, respiratory insufficiency, peripheral arterial occlusive disease, obesity)^4,5^. Cerebral palsy, as a group of neurological disorders, affects about 2 in every 1000 newborns. Its prevalence reaches 5–8% among newborns with very low birth weights or very pre-term deliveries. Gait disturbances have a tremendous impact on patients, especially on their quality of life^1^: they complain most often of pain, joint stiffness, numbness, or weakness^6^. Neurological gait disorders, in particular, are associated with lower cognitive function, depressed mood, and diminished quality of life^7^. To have insight into patients’ conditions and therefore treat their gait disorders, clinicians historically used Observational Gait Analysis (OGA)^8^. OGA usually relies on a clinician’s observation freeze-framed techniques and video slow-motion replay to record and analyze a patient’s gait. It is subject to bias and has limited precision because it relies on the experience of the clinician. To overcome this limitation, Quantitative Gait Analysis (QGA) is considered. It uses instrumentation to quantify the gait cycle by recording temporal-spatial, kinematic, and kinetic data that is rarely gathered by observation. The challenge facing clinicians is to analyze a large amount of clinical data from QGA in order to determine the severity of the illness and select the most effective therapeutic strategy. It is a very tricky task because of the great disparity between patients (e.g., children and adults) and the diversity of their pathologies. In this context, our aim is to assist clinicians in analyzing this large amount of clinical data with an artificial intelligence applied to kinematics from QGA. The target objective is to go beyond objectively quantifying gait quality by predicting whether it will improve within the next visit. These predictions tend to help clinicians select the most effective treatment strategy. For this purpose, two approaches were considered: signal-based, which uses raw gait cycles, and image-based, which converts gait cycles into image-like representations, making them suitable for training image-based deep neural networks, especially pre-trained ones. In the signal-based approach, a Long Short Term Memory (LSTM) and a MultiLayer Perceptron (MLP) were designed from scratch. Their hyper-parameters were tuned with KerasTuner^9^. The obtained results were compared to five state-of-the-art architectures^10^, including Fully Convolutional neural Network (FCN), Residual Network (ResNet), Encoder, Time Le-Net (t-LeNet), and Transformer. For the two tailored and state-of-the-art architectures, the influence of data augmentation was studied. In the image-based approach, the first step was to map the time representation of 1D gait cycles to a 2D frequency representation using the two-dimensional Fast Fourier Transform (2D FFT). Then, the obtained 2D FFT images were processed with four pre-trained Convolutional Neural Networks (CNN): VGG16, ResNet34, EfficientNet_b0, and a Vision Transformer (ViT). The obtained results were compared to those of a tailored CNN with a much smaller number of parameters. The effectiveness of the proposed models was evaluated on a gait dataset collected from more than 700 patients.

## Materials and methods

### Data acquisition

This study was carried out in accordance with the tenets of the Declaration of Helsinki and with the approval of the Brest, France hospital’s (CHRU’s) Ethics Committee. Patients had also signed an informed consent. Our work was conducted between 2021 and 2022. Data collected between June 2006 and June 2021 from 734 patients (115 adults and 619 children) who had undergone clinical 3D gait analysis were used. Their identities were preserved by respecting medical secret and protecting patient confidentiality. All data were recorded using the same motion analysis system (Vicon MX, Oxford Metrics, UK) and four force platforms (Advanced Mechanical Technology, Inc., Watertown, MA, USA) in the same motion laboratory (CHU Brest) between 2006 and 2022. The data collected by the 15 infrared cameras (sampling rate of 100 or 120 Hz) were synchronized with the ground reaction forces recorded by the force platforms (1000 Hz or 1200 Hz). The 16 markers were placed according to the protocol by Kadaba et al.^11^. Marker trajectories and ground reaction forces were dual-pass filtered with a low-pass Butterworth filter at a cut-off frequency of 6 Hz. After an initial calibration in the standing position, all patients were asked to walk at a self-selected speed along a 10m walkway.

Gait kinematics were processed using the Vicon Plug-in Gait model. Kinematics were time-normalized to stride duration, from 0 to 100% from initial contact (IC) to the next IC of the ipsilateral foot. Nine gait joint angles (kinematic gait variables) were used: anteversion/retroversion of the pelvis, rotation of the pelvis, pelvic tilt, flexion/extension of the hip, abduction/adduction of the hip, internal/external rotation of the hip, flexion/extension of the knee, plantar/dorsiflexion of the ankle, and the foot’s angle of progression. As a result, a gait cycle yielded 101 $\times$ 9 measurements. Let $E_{p,d}$ denote the gait session of patient *p* at datetime *d*. It can be written as follows:

1 $$\begin{aligned} E_{p,d} = \left\{ {C_{ E_{p,d}}}^{1}, {C_{ E_{p,d}}}^{2}, \ldots , {C_{ E_{p,d}}}^{K} \right\} \end{aligned}$$

where ${C_{ E_{p,d}}}^{k}$ is the *k*-th gait cycle of a gait session $E_{p,d}$ and *K* the total number of gait cycles. Let $c_{t,n}^{E_{p,d}^{k}}$ denote the gait cycle ${C_{E_{p,d}}}^{k}$ value at time step *t* and joint angle *n*. To keep notations simple, $c_{t,n}^{E_{p,d}^{k}}$ is referred to as $c_{t,n}$ in what follows. ${C_{E_{p,d}}}^{k}$ can simply be represented with a matrix of 101 lines and 9 columns, as follows:

2 $$\begin{aligned} {C_{ E_{p,d}}}^{k} = \begin{bmatrix} c_{1,1} &{} c_{1,2} &{}\cdots &{} c_{1,9} \\ c_{2,1} &{} c_{2,2} &{}\cdots &{} c_{2,9}\\ \vdots &{} &{} &{} \\ c_{101,1} &{} c_{101,2} &{}\cdots &{} c_{101,9}\\ \end{bmatrix} \end{aligned}$$

The Gait Profile Score (GPS), a “walking behavior score”, was computed for each gait cycle from the previously described joint angles^12–14^. The GPS is a single index measure that summarizes the overall deviation of kinematic gait data relative to normative data. It can be decomposed to provide Gait Variable Scores (GVS) for nine key component kinematic gait variables, which are presented as a Movement Analysis Profile (MAP). The GVS corresponding to the *n*-th kinematic variable, GVS$_{\textrm{n}}$, is given by^15–17^:

3 $$\begin{aligned} GVS_n = \sqrt{\frac{1}{T}\sum _{t=1}^{T}(c_{t,n} - c_{t,n} ^{ref})^{2}} \end{aligned}$$

where *t* is a specific point in the gait cycle, *T* its total number of points (typically equal to 101^18,19^), $c_{t,n}$ the value of the kinematic variable *n* at point *t*, and $c_{t,n}^{\textrm{ref}}$ is its mean on the reference population (physiological normative). The GPS is obtained from the GVS scores^15,17^ as follows:

4 $$\begin{aligned} GPS = \sqrt{\frac{1}{N}\sum _{n=1}^{N}GVS_n^{2}} \end{aligned}$$

where *N* is the total number of kinematic variables (equal to 9 by definition).

### Gait database

**Figure 1:**
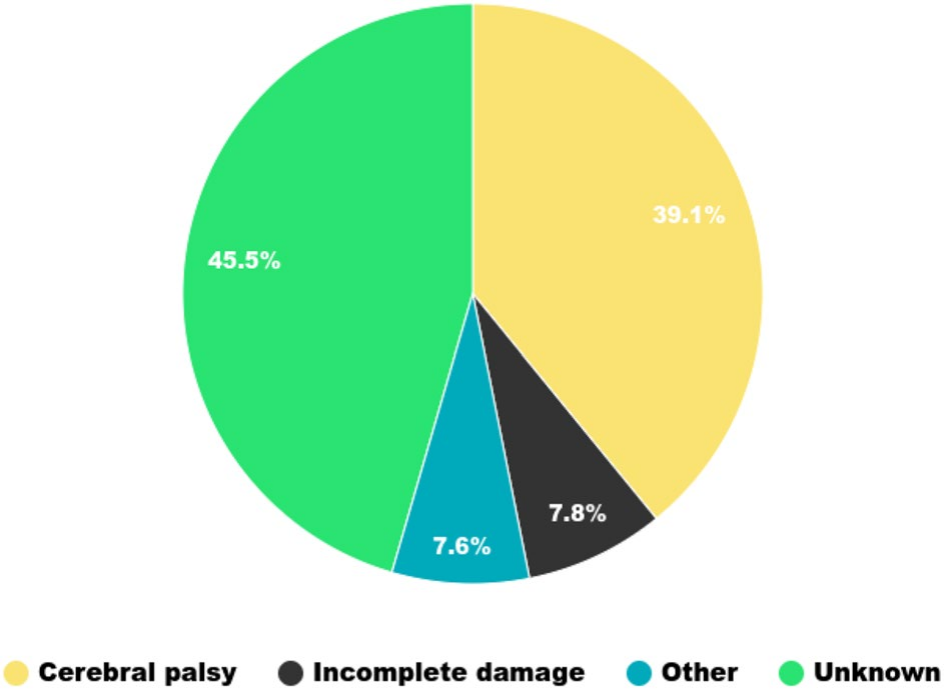
Etiologies pie chart.

We had a total of 1459 gait sessions from 734 patients (115 adults and 619 children). Each patient had an average of 1.988 gait sessions with a standard deviation of 1.515. 53,693 gait cycles were collected. Their average number per gait session is equal to 18 with a standard deviation of 6. Neurological conditions, notably cerebral palsy, are the most frequent etiologies, as we can see in Fig. 1.

The average patient age within the first gait session is equal to 14 years, with a standard deviation of 16 years. The time delay between the first and last gait session (for patients with more than one gait session, i.e., 319) is equal to 3.92 years on average with a standard deviation of 3.24 years. Directly consecutive gait sessions are, on average, separated by approximately 740 days, with a standard deviation of 577 days. The shortest (resp. longest) time delay was equal to 4 (resp. 4438) days. We had 1384 pairs of directly consecutive gait sessions belonging to 319 patients (the remaining patients were removed since they had only one gait session). Involved gait conditions are various: without any equipment, with a cane, with a rollator, with an orthosis, with a prosthesis.. Only pairs of gait sessions without equipment were selected in order to be in the same condition (79% of all available pairs, i.e. 1152). The first gait sessions in these pairs were used for training. Models were fed the gait cycles of these first gait sessions (i.e., 21,167 gait cycles in total).

### GPS variation prediction

GPS variation prediction is similar enough to a Time Series Classification (TSC) issue that its proposed popular architectures should be adopted. Consecutive gait session pairs $(E_{p,d}, E_{p,d+\Delta d})$ were considered. For each gait cycle ${C_{ E_{p,d}}}^{k}$ of the current gait session $E_{p,d}$, a GPS variation $\Delta {}GPS$ was computed using:

5 $$\begin{aligned} \Delta {}GPS({C_{ E_{p,d}}}^{k}) = GPS_{avg}( E_{p,d+\Delta d}) - GPS({C_{ E_{p,d}}}^{k}) \end{aligned}$$

where $GPS_{avg}(E_{p,d+\Delta d})$ is the average GPS per cycle of $E_{p,d+\Delta d}$ and $GPS({C_{ E_{p,d}}}^{k})$ the GPS of the current gait cycle ${C_{E_{p,d}}}^{k}$. The average GPS per cycle $GPS_{average}(E_{p,d})$ of a gait session $E_{p,d}$ is simply equal to:

6 $$\begin{aligned} GPS_{avg}(E_{p,d}) = \frac{\sum _{k=1}^{K} GPS({C_{ E_{p,d}}}^{k}) }{K} \end{aligned}$$

$$\Delta {}$$

GPS was ranked in a binary fashion. Either it is negative, in which case the patient’s gait improves (class 1), or it is positive, in which case the patient’s gait worsens (class 0). The metric used is the Area Under the Curve (AUC).

The distribution of patients between training, validation, and test groups is provided in Table 1. Such a split put 73%, 12%, and 14% of total gait cycles within the training, validation, and test groups, respectively.

**Table 1:** Data distribution for $\Delta {}$GPS prediction.

Train	Validation	Test
224 patients	48 patients	47 patients
15509 cycles	2678 cycles	2980 cycles
844 gait session pairs	142 gait sessions pairs	166 gait session pairs
45.61 % of class 0	45.89 % of class 0	44.4% of class 0

#### Signal-based approach

To be exhaustive, one MLP, one recurrent neural network (LSTM), one hybrid architecture (Encoder), several CNN architectures (FCN, ResNet, t-LeNet), and a one-dimensional Transformer^20^ were included. The MLP and LSTM were designed and developed from scratch. Their hyper-parameters were optimized manually. FCN, ResNet, Encoder, and t-LeNet are among the most effective end-to-end discriminative architectures regarding the TSC state-of-the-art^10^. These methods were also compared to the Transformer, a more recent and popular architecture. The Transformer does not suffer from long-range context dependency issues compared to LSTM^21^. In addition, it is notable for requiring less training. The Adam optimizer^22^ and binary cross-entropy loss were employed^23^.

**Figure 2:**
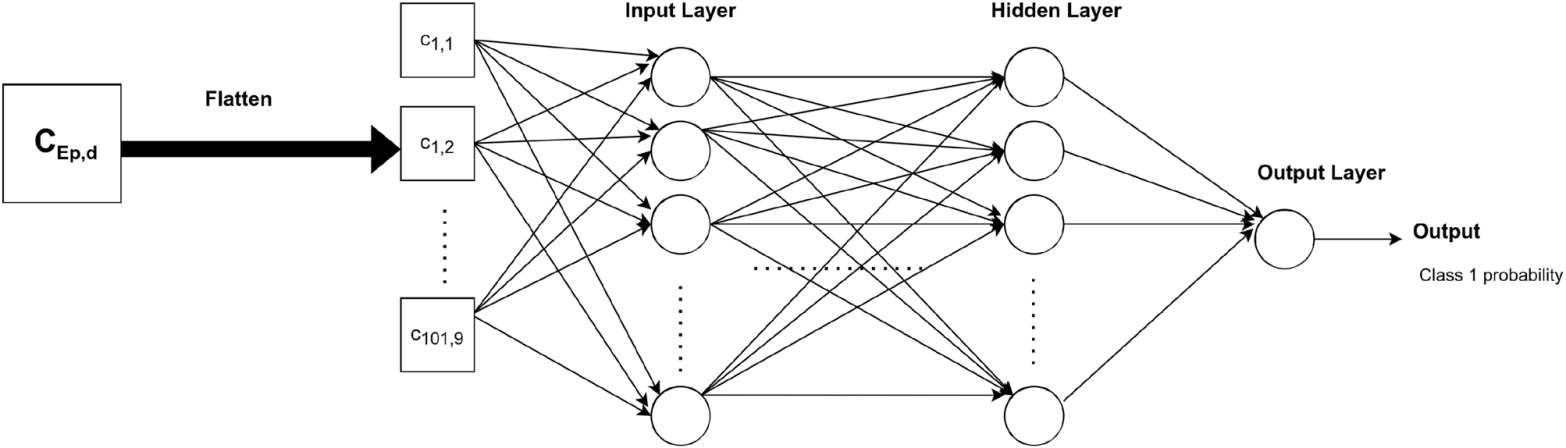
MLP architecture for prediction.

For MLP, gait cycles were flattened so that the input length was equal to 909 time steps. The number of neurons was the same across all the fully connected layers. Many values of this number were tested to find the best structure for our task. In the same way, the number of layers was optimized. The corresponding architecture is shown in Fig. 2.

**Figure 3:**
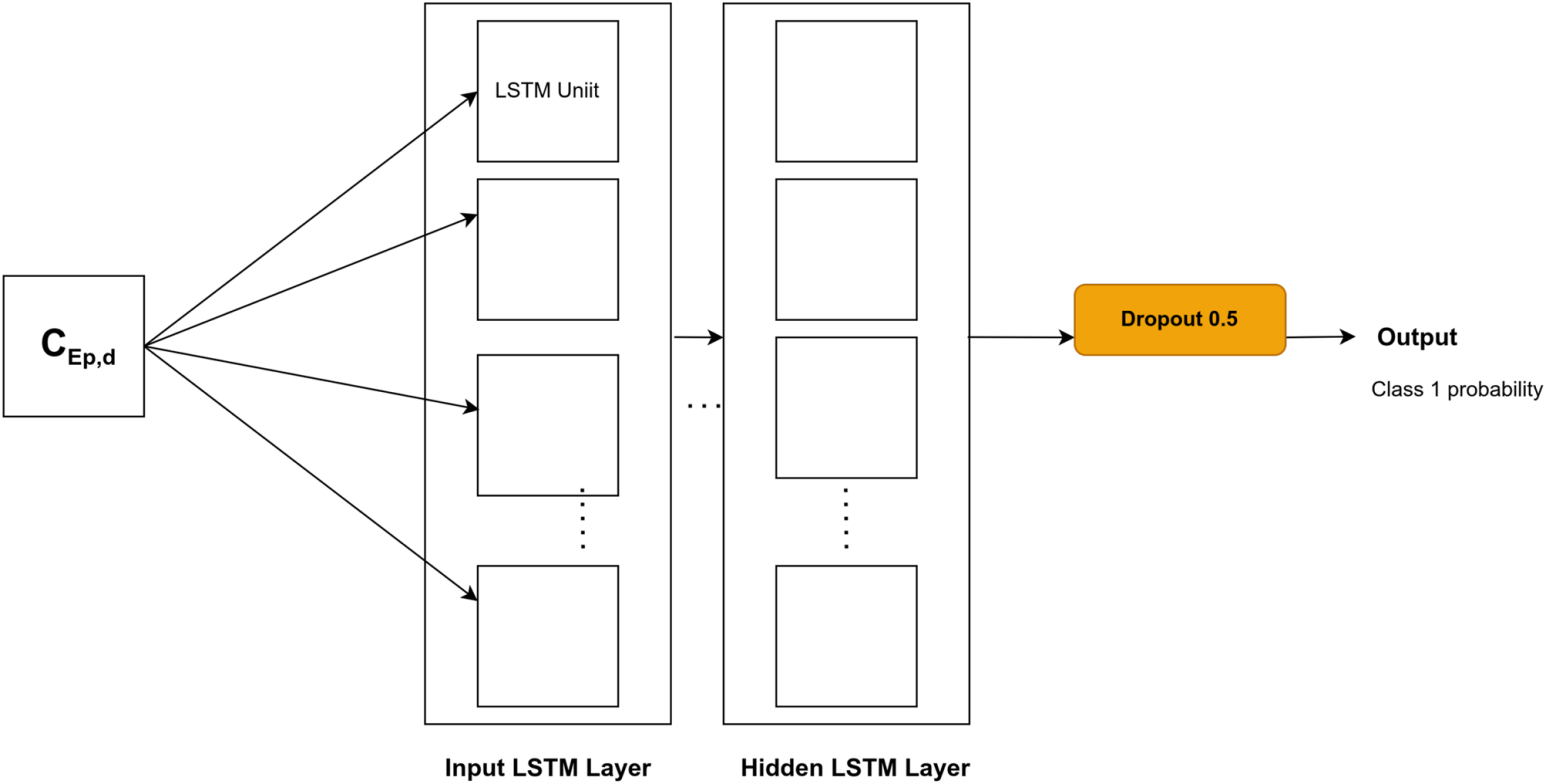
LSTM architecture for prediction.

LSTM layers were stacked, and a dropout was added before the last layer to avoid overfitting. The corresponding architecture is shown in Fig. 3.

**Figure 4:**
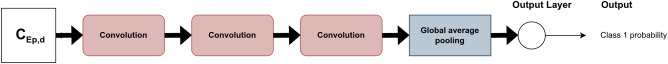
FCN architecture for prediction.

**Figure 5:**
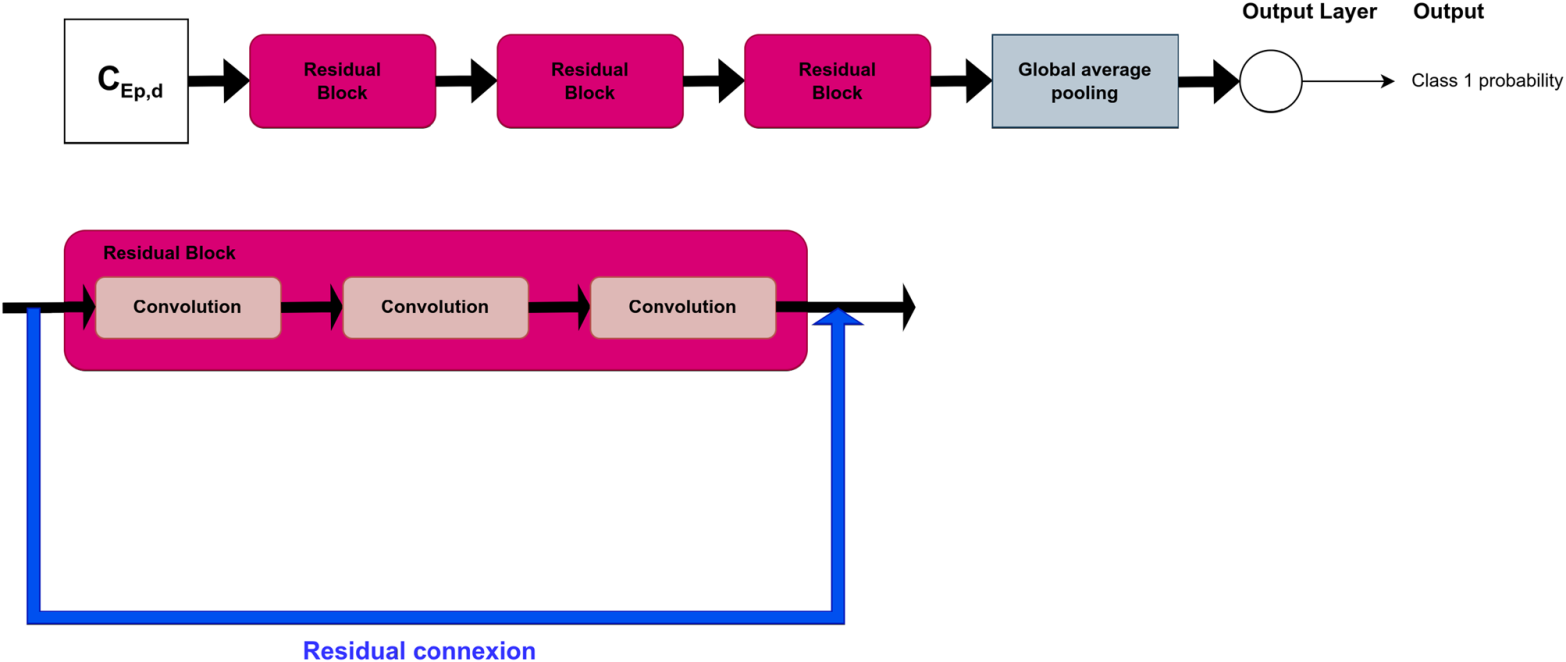
ResNet architecture for prediction.

**Figure 6:**
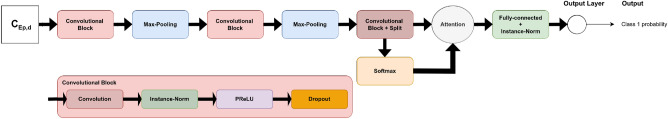
Encoder architecture for prediction.

**Figure 7:**
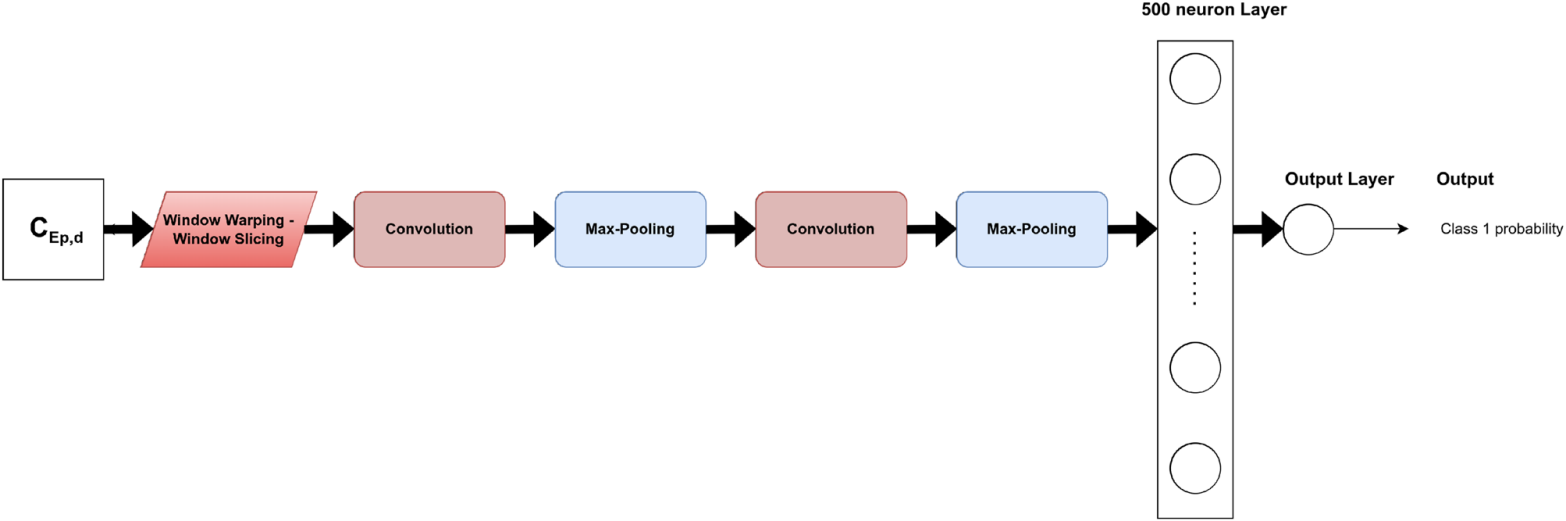
t-LeNet architecture for prediction.

For FCN, ResNet, Encoder and t-LeNet, the architectures proposed in Ref.^10^ were considered. They are shown in Figs. 4, 5, 6 and 7, respectively. We followed an existing implementation^24^ to set up the Transformer.

### Data augmentation

Different techniques of data augmentation were tested as a pre-processing step to avoid overfitting: jittering, scaling, window warping, permutation, and window slicing. Their hyperparameters were empirically optimized for each model. These are among the TSC literature’s most frequently utilized techniques, particularly when it comes from sensor data^10^.

#### Image-based approach

Image-based time series representation initiated a new branch of deep learning approaches that consider image transformation as an innovative pre-processing of feature engineering^25^. In an attempt to reveal features and patterns less visible in the one-dimensional sequence of the original time series, many transformation methods were developed to encode time series as input images.

**Figure 8:**
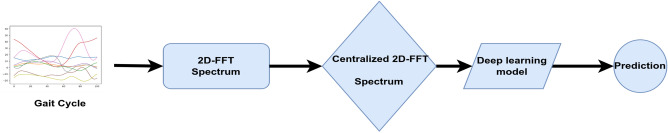
Proposed $\Delta GPS$ prediction workflow for the image-based approach.

In our study, sensor modalities are transformed to the visual domain using 2D FFT in order to utilize a set of pre-trained CNN models for transfer learning on the converted imagery data. The full workflow of our framework is represented in Fig. 8.

**Figure 9:**
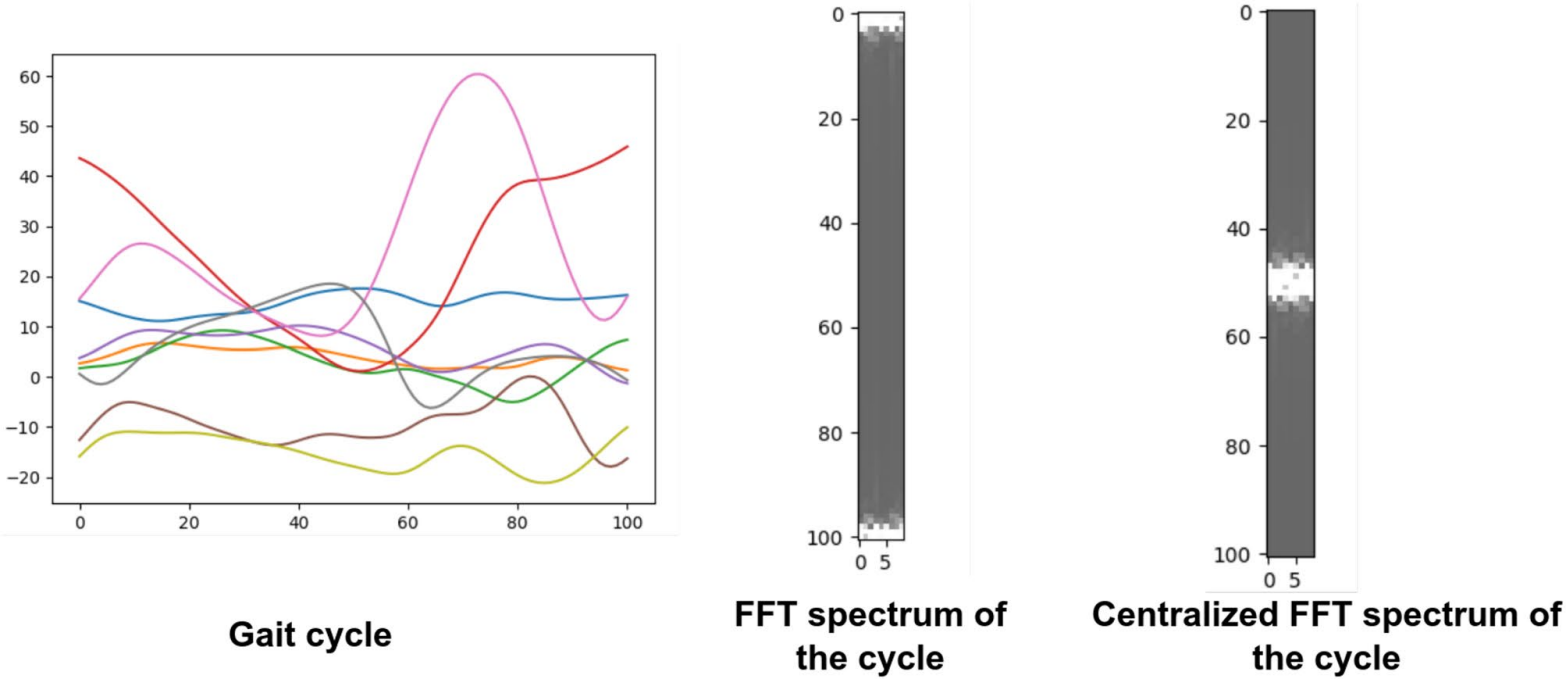
2D FFT for a given gait cycle. (**a**) The gait cycle; (**b**) FFT spectrum of the gait cycle; (**c**) Centralized FFT spectrum of the gait cycle.

2D FFT is used to work in the frequency domain or Fourier domain because it efficiently extracts features based on the frequency of each time step in the time series. It can be defined as:

7 $$F(u,v) = \frac{1}{{T.N}}\sum\limits_{{t = 0}}^{T} {\sum\limits_{{n = 0}}^{N} {c_{{t,n}} } } \exp \left( { - j2\pi \left( {\frac{{ut}}{T} + \frac{{vn}}{N}} \right)} \right)$$

where *F*(*u*, *v*) is the direct Fourier transform of the gait cycle. It is a complex function that shows the phase and magnitude of the signal in the frequency domain. *u* and *v* are the frequency space coordinates. The magnitude of the 2D FFT |*F*(*u*, *v*)|, also known as the spectrum, is a two-dimensional signal that represents frequency information. Because the 2D FFT has translation and rotation attributes, the zero-frequency component can be moved to the center of |*F*(*u*, *v*)| without losing any information, making the spectrum image more visible. The centralized FFT spectrums were computed and fed to the proposed deep learning models. A centralized FFT spectrum for a given gait cycle is represented in Fig. 9.

### Proposed deep learning models

#### Timm pre-trained models

The Timm library’s^26^ pre-trained VGG16, ResNet34, EfficientNet_b0, and the Vision Transformer ’vit_base_patch16_224’ were investigated. They were pre-trained on a large collection of images, in a supervised fashion. For the Transformer, the pre-training was at a resolution of $224 \times 224$ pixels. Its input images were considered as a sequence of fixed-size patches (resolution $16 \times 16$), which were linearly embedded.

Converting our grayscale images to RGB images was not necessary because Timm’s implementations support any number of input channels. The model’s minimum input size for VGG16 is $32 \times 32$. The image’s width dimension (N) equals 9, which is less than 32. In order to fit the minimum needed size, 2D FFT images were repeated 4 times in this width dimension. Transfer learning with fine-tuning methods was employed. One neuron’s final fully connected layer was used. In the same way that the top layers were trainable, all convolutional blocks were.

#### Two-dimensional 2D CNN

**Figure 10:**
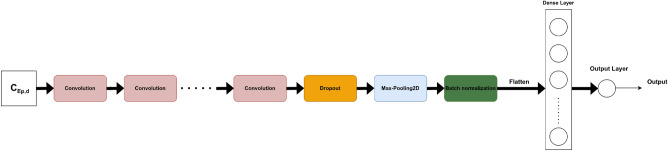
Tailored 2D CNN for prediction.

The pre-trained Timm models are deep and sophisticated, with many layers. As a result, a CNN model with fewer parameters, designed from scratch, was conceived. The number of used two-dimensional convolutional layers was a hyper-parameter to optimize in a finite range of values {1, 2, 3, 4, 5}. After the convolutional block, a dropout function was applied. Following that, two-dimensional max-pooling (MaxPooling2D) and batch normalization were used. The flattened output of the batch normalization was then fed to a dense layer of a certain number of neurons to tune. In order to predict the $\Delta GPS$, our model had a dense output layer with a single neuron. The corresponding architecture is shown in Fig. 10.

The following are all of the architecture hyper-parameters to tune: the number of convolutional layers (num_layers), the number of filters for each convolution layer (num_filters), the kernel size of each convolution layer (kernel_size), the dropout rate (dropout), the pooling size of the MaxPooling2D (pool_size), the number of neurons in the dense layer (units), and the learning rate (lr). Five models with a varying number of convolutional layers (from 1 to 5) were tested. For each of them, the rest of the hyper-parameters were tuned using KerasTuner^9^ to maximize the validation AUC.

## Results

In this section, prediction results are presented in terms of AUC.

### Signal-based approach

#### Without data augmentation

Results are given in Table 2. They are homogeneous on the validation set. LSTM and MLP perform equally well on the validation set. ResNet has the highest val AUC (0.709) for the state-of-the-art architectures. FCN achieves a comparable result to ResNet with a val AUC of 0.705. Encoder, t-LeNet and Transformer perform nearly equally well, with a val AUC above 0.63.

**Table 2:** GPS variation prediction results. Best results in bold.

Model	Epoch of convergence	Val AUC	Test AUC	Test accuracy
MLP	55	**0.717**	**0.683**	**0.640**
LSTM	3	0.701	0.710	0.665
FCN^10^	30	0.705	0.689	0.641
ResNet^10^	8	0.709	0.699	0.663
Encoder^10^	25	0.631	0.691	0.648
t-LeNet^10^	5	0.653	0.689	0.64
Transformer^20^	851	0.640	0.709	0.626

### MLP

The best model has 4 layers of 200 neurons each. It is referred to as MLP_4_200. It gives a val AUC equal to 0.717.

### LSTM

The best model has 4 LSTM layers of 500 units. It is referred to as LSTM_4_500. It gives a val AUC of 0.701.

#### Data augmentation

For all the architectures used, an overfitting behavior with very quick convergence was exhibited. To mitigate this, 5 data augmentation techniques already presented were combined. The order of their application was chosen randomly for each training batch. The best data augmentation parameters were found for each architecture. Results are presented in Table 3. Performances are slightly better after data augmentation except for the Transformer. In general, convergence is slower; it no longer appears in the first few epochs. FCN gives the best val AUC (0.723).

**Table 3:** Prediction results after data augmentation.

Model	Epoch of convergence	val AUC	test AUC	Test accuracy
MLP_4_200	2932	0.667	0.667	0.643
LSTM_4_500	2126	0.712	0.710	0.66
FCN^10^	1517	**0.723**	**0.717**	**0.598**
ResNet^10^	172	0.719	0.716	0.663
Encoder^10^	1942	0.640	0.632	0.604
t-LeNet^10^	61	0.625	0.665	0.618
Transformer^20^	322	0.546	0.561	0.467

Significant values are in bold.

### Image-based approach

Table 4 presents the obtained results.

**Table 4:** Quantitative results. Best results in bold.

Architecture	Number of parameters	val AUC	test AUC
VGG16	134,263,489	0.650	0.642
ResNet34	21,278,913	0.653	0.679
EfficientNet_b0	4,008,253	0.637	0.628
Vision Transformer	85,260,289	0.652	0.691
CNN	35,505	**0.726**	**0.693**

Despite the overfitting behavior of the pre-trained models, the test set’s results are nearly identical to those from the validation set. The tested Timm models all produced results that were comparable, with a val AUC of greater than 0.63. The model with the highest efficiency, the CNN trained entirely from scratch, gives a val AUC of 0.726. It has two convolutional layers, and its hyper-parameter values are as follows: num_filters = 4, kernel_size = 32, dropout = 0, pool_size = 8, units = 300 and lr = $4.127 \times 10^{-4}$. Transfer learning is unlikely to have made a significant contribution because our images are visually insufficiently meaningful. Besides, there are not enough large datasets (from the same domain) available within the community to carry out such a transfer learning task.

## Discussion and conclusion

**Figure 11:**
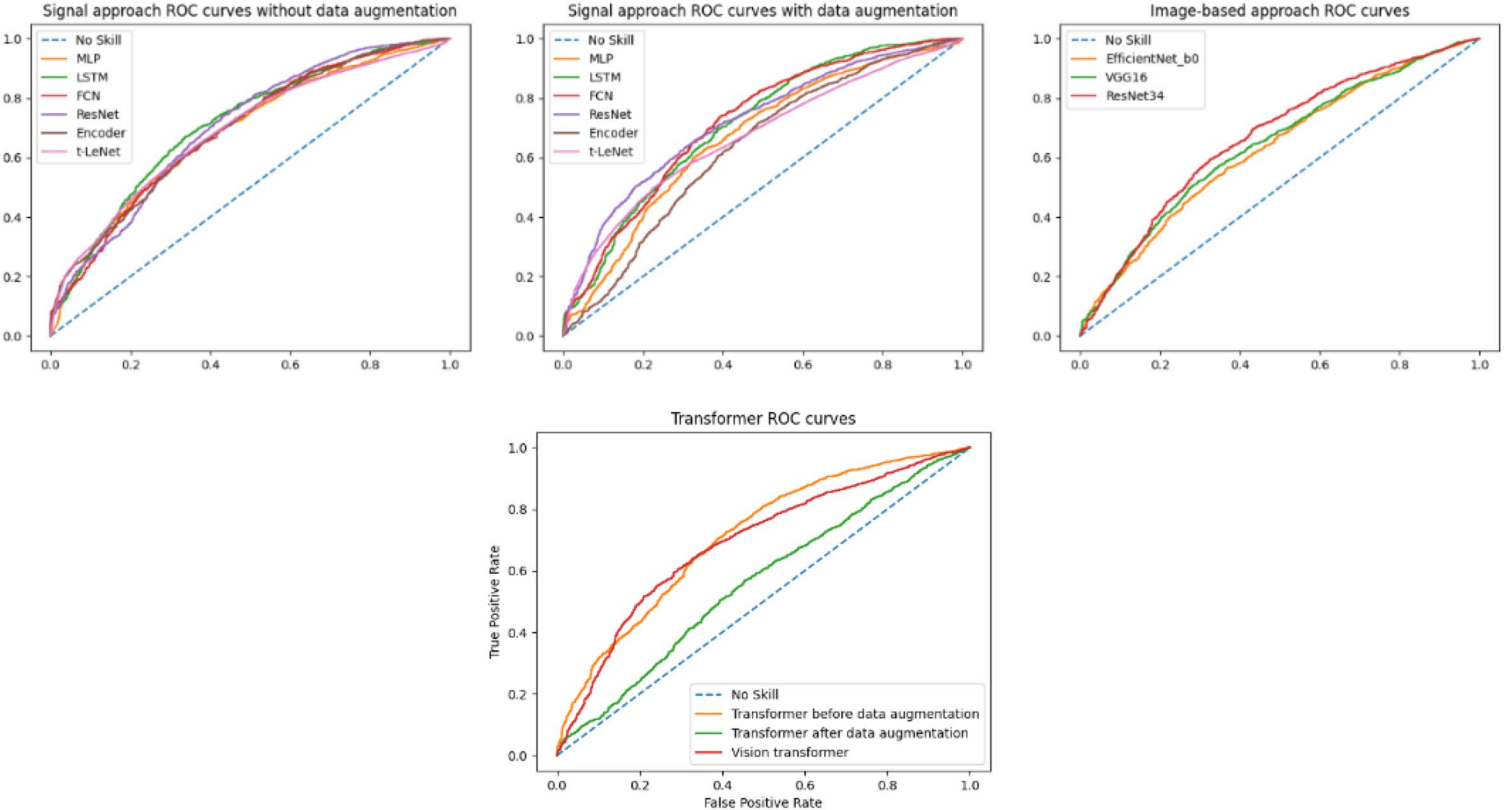
ROC curves for both approaches.

The goal of our study was to predict the $\Delta GPS$ between two consecutive gait sessions in a binary fashion. If this variation is negative, gait quality gets better and vice versa. Globally, from scratch designed architectures gave slightly better results than state-of-the-art ones, which introduce too many parameters to optimize given the relatively small quantity of available data. As a result, a trade-off should be made between the amount of available training data, the complexity of the task, and performance. In the signal-based approach, in general, data augmentation techniques made some improvements in performance. Because of that, we suggest trying to find a way to improve their efficiency. In the image-based approach, developed from scratch CNN surpassed pre-trained Timm models. This can be explained by the fact that the source and target domains are so different. ROC (Receiver operating characteristic) curves for all models are presented in Fig. 11.

To have better insight into results, the ROC curves of the best models (i.e., FCN after data augmentation for the signal-based approach and CNN for the image-based one) were compared using the DeLong’s test. This revealed a p-value of $2.316 \times 10^{-4}$ for the two ROC curves at hand, which means that the AUCs of both models are significantly different. In other words, the FCN model after data augmentation, with a val AUC of 0.723 and a test AUC of 0.717, is meaningfully better than the CNN model. This outcome proves that knowledge extraction is more efficient on raw signals than synthetic images. In summary, for both approaches, the prediction results are encouraging despite the complexity of such a prediction task on so heterogeneous data. The val AUC and test AUC are above 0.7 for both approaches.

One limitation of this study is the fact that we were not able to validate our findings on external datasets because we did not have any other external data at our disposal. Actually, we were unable to find any publicly-available medical databases.

Our future work will focus on taking the different pathologies into account. Ways of having more data should be thought about as well.

## Data Availability

The dataset used and analysed during the current study is available from the corresponding author on reasonable request.
